# Circuit analogy unveiled the haemodynamic effects of the posterior cross vein in the wing vein networks

**DOI:** 10.1371/journal.pone.0301030

**Published:** 2024-04-02

**Authors:** Kazuki Sugiyama, Yoshihiro Kubota, Osamu Mochizuki

**Affiliations:** 1 Graduate School of Science and Engineering, Toyo University, Saitama, Japan; 2 Department of Mechanical Engineering, Toyo University, Saitama, Japan; University of Birmingham, UNITED KINGDOM

## Abstract

We investigated the wing vein network topology in fruit flies and observed that the posterior cross vein (PCV) disrupts the symmetry of the entire network. The fluidic engineering function of this vein’s disposition remains unexplored although the wing vein network is known to transport blood. We examined the fluid mechanical effects of the PCV’s disposition on this blood-transporting network through numerical simulations involving the removal and rearrangement of the vein, avoiding impractical physical manipulation. We characterised the geometry of each wing membrane cell, a portion of the wing membrane surrounded by a group of veins, by determining the ratio of its surface area to the contact area with the veins. We considered this ratio in association with the flow velocities of seeping water from the blood within the veins to the membrane and evaporating water from the membrane, based on the mass conservation law. We observed that the division of a membrane cell by the PCV maximises the ratio of the areas in the divided cell on the wing-tip side by virtually shifting this vein’s connections in our geometric membrane model. We derived blood flow rate and pressure loss within the venous network from their geometry, using an analogy of the venous network with a circuit consisting of hydraulic resistors based on Kirchhoff and Ohm’s laws. The overall pressure loss in the network decreased by 20% with the presence of the PCV functioning as a paralleled hydraulic resistor. By contrast, any other cross-vein computationally arranged on another membrane cell as the PCV’s substitution did not exhibit a larger reduction in the pressure loss. Overall, our numerical analyses, leveraging geometry and a circuit analogy, highlighted the effects of the PCV’s presence and position on the blood-transporting vein network.

## Introduction

The vein networks on insect wings transport insect blood, haemolymph, which is associated with the vital supply to the insect wings. In most winged insects, haemolymph enters the wing from the anterior vein at its base, flows across the entire vein network, and exits from the posterior vein at its base [1–3], aspirated by accessory pulsatile organs [4–6]. The internal haemolymph is considered to serve vital substances to living tissues in the wing [6] and provide hydration that maintains mechanical properties of the wing [7, 8]. Therefore, an insect wing employs a fluid supply system consisting of conduits and a pump. Although previous studies revealed the resultant flow routes and physiological importance of haemolymph circulation, its physical aspects for the vital supply, such as flow rate and pressure loss, remain unknown.

The structure of the vein network should be one of the factors dominating the internal haemolymph flow. Because the wing veins are fluid-transporting conduits, we can consider them as hydraulic resistors forming a circuit. From an engineering viewpoint, reduction in the net hydraulic resistance of the circuit is advantageous to improve pressure drop and power consumption to maintain flow. A previous study has suggested that the branching architectures of wing veins of butterfly and moth are adjusted to minimise total power consumptions due to pressure drop and metabolic activities by demonstrating that vein diameters at some bifurcations in these insect wing venations follow Murray’s law [9]. Evolutionary adjustment in the entire network structures might also contribute to improvement of haemolymph circulation. However, their haemodynamic effect has not been well explored. The understanding of network adjustment mechanism for low-energy consuming fluid transportation in the insect wing veins not only provides insight into relationship between the venation morphology and circulatory physiology of the insect wing, but also inspires network design for efficient microfluidic devices.

**Fig 1:**
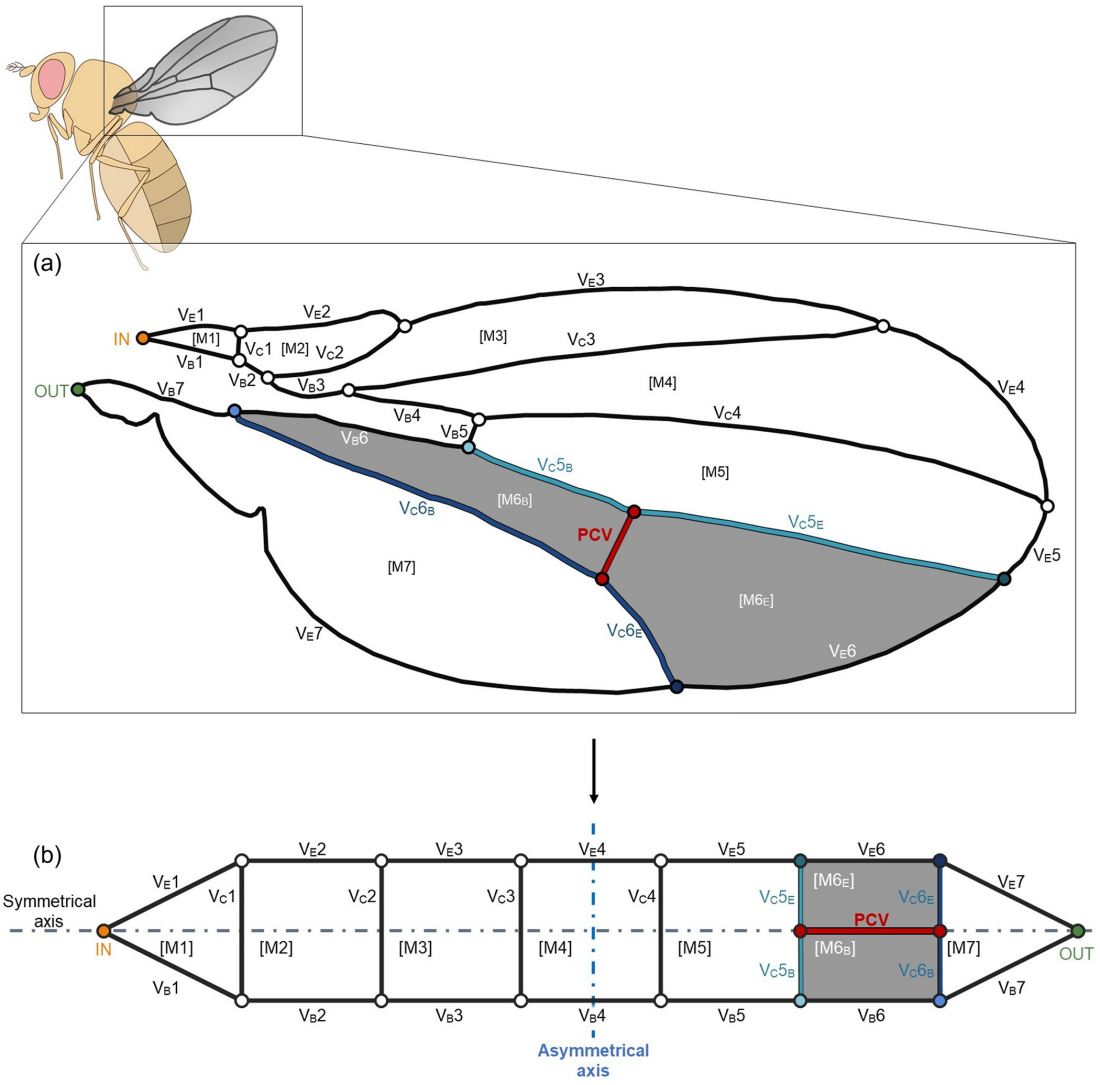
Characterisation of the wing vein network through topological transformation. (a) Schematic of the wing venation in *D*. *melanogaster*. (b) Topologically transformed vein network. The black or coloured lines denote the wing veins, and circles indicate the bifurcations of the veins. The veins with numbers starting with “V_E_” and “V_B_” are the edge veins and base veins, respectively. “V_C_” represents the connecting veins. The light blue and dark blue veins are separated into edge- and base-side segments that are respectively subscripted with “E” and “B”. The red vein that is captioned “PCV” is the posterior cross vein (PCV). The numbers starting with “M” in brackets represent the membrane cells. The grey-coloured cells with subscripted “E” and “B” indicate the edge- and base-side portions of the membrane cell divided by the PCV. The circles at both ends of the network captioned “IN” and “OUT” denote the inlet and outlet of haemolymph flow.

We targeted the wing vein network structure of the forewings of the fruit flies, *Drosophila melanogaster*, to demonstrate its haemodynamic impact. This network was characterised through topological transformation based on the graph theory, as shown in Fig 1. Furthermore, the posterior cross vein (PCV) that prevents the formation of a perfectly symmetrical ladder structure was differentiated, as highlighted in red in Fig 1b. This vein is commonly found in the species of *Drosophila* genus [10]. It emerges during a specific developmental process following the formation of most other veins [11, 12], and its position varies among species [13]. Despite its prevalence, its function has not been gained attention from engineering viewpoint, and its impact on the haemolymph circulation remains unknown.

In this study, we focused on the haemodynamic effect of the PCV from a fluid mechanical perspective. We needed to compare the haemodynamics in the wing vein networks in its absence and presence to demonstrate the effect of this vein. Its positional effect was investigated by rearranging it. Observation-based experiments such as those of traditional studies may pose challenges in physical manipulation of the wing veins in a living insect. It causes detrimental degree of complexity that might be the reason for the scarce knowledge about the fluidic impact of the wing vein networks. Instead, this study utilised numerical approaches to virtually remove or rearrange the PCV for validation of this vein’s effect. We characterised the geometry of the wing membrane cells, portions of the wing membrane surrounded by a group of veins, to assess the influence of the PCV on the division of specific membrane cells. We employed the analogy of the wing vein network as a circuit composed of hydraulic resistors and calculated the volumetric haemolymph flow rate and pressure loss in the venous network. This study demonstrates the fluid mechanical effects of the PCV on haemolymph circulation in the wing vein network of the fruit fly by integrating these results.

## Materials and method

### Characterisation of the fruit fly’s wing

We characterised the vein network in the forewing of the fruit fly, *D*.*melanogaster*. Certain veins follow the edge of the wing (V_E_1–7), whereas others run along the base (V_B_1–7), as illustrated in Fig 1a. We named these sets the edge veins and the base veins, respectively. In our topological model (Fig 1b), these vein sets correspond to the upper and lower serial veins, connected by veins denoted as V_C_1–6 in Fig 1a, which are named the connecting veins. The PCV, highlighted in red, links two of the connecting veins. This vein divides a specific longitudinal membrane region in the wing, coloured in grey in Fig 1a, resulting in distinct morphologies of the separated cells.

To characterise the geometry of the wing membrane cells, we determined the ratio of the surface area to the contact area with their surrounding veins. The total surface area of the membrane cell was defined as the area exposed to the atmosphere, *A*_S_ = 2*A*_m_, where *A*_m_ is the area of each side of the membrane. The contact area with each vein was defined as half of the vein wall area, given that a membrane cell contacts one side of the vein. This contact area is expressed as *πl*_n_*d*_n_ /2, where *l*_n_ and *d*_n_ are the length and inner diameter of each vein, respectively. The total contact area of a membrane cell with its surrounding veins is expressed as *A*_C_ = Σ*πl*_n_*d*_n_ /2. We determined the balance between the surface area and the contact area with their surrounding veins using the calculated values of *A*_S_ /*A*_C_.

The geometric parameters of the wing, including *A*_m_, *l*_n_, and *d*_n_, were obtained by analysing a digital photograph of the forewing of *D*. *melanogaster* [14] using ImageJ software (courtesy of the National Institutes of Health, United States of America) with the minimal resolution of 2.5 μm/pixel. The length of each vein was defined as the average of three measurements, whereas the outer diameter was defined as the average of measurements at five different sections of each vein. The area of each membrane cell was defined as a two-dimensional area surrounded by a group of veins. The obtained parameters are listed in Tables 1 and 2. We treated the cross-section of the haemolymph-filled space as circular, and its diameter *d*_n_ was assumed to be equal to 20% of the measured outer diameter, based on the cross-sectional image of the vein in *D*.*melanogaster* [15]. The vein lengths and diameters may respectively contain 0.92% and 15% of errors in average owing to the spatial resolution for the image analysis.

**Table 1:** Geometries of the wing veins of *D*. *melanogaster*.

Vein	Length: *l*_n_ [μm]		Outer diameter [μm]		Inner diameter: *d*_n_ [μm]
V_E_1	3.0 × 10^2^	±14	2.8 × 10^1^	±11	5.6 × 10^0^
V_E_2	4.2 × 10^2^	±0.96	2.2 × 10^1^	±5.9	4.4 × 10^0^
V_E_3	1.2 × 10^3^	±4.7	2.3 × 10^1^	±8.7	4.5 × 10^0^
V_E_4	6.4 × 10^2^	±2.8	1.5 × 10^1^	±1.8	3.1 × 10^0^
V_E_5	2.2 × 10^2^	±0.42	9.4 × 10^0^	±1.9	1.9 × 10^0^
V_E_6	8.7 × 10^2^	±1.8	8.7 × 10^0^	±1.2	1.7 × 10^0^
V_E_7	1.9 × 10^3^	±11	1.1 × 10^1^	±5.5	2.2 × 10^0^
V_B_1	3.1 × 10^2^	±3.0	6.0 × 10^1^	±5.3	1.2 × 10^1^
V_B_2	8.7 × 10^1^	±4.9	4.9 × 10^1^	±1.9	9.9 × 10^0^
V_B_3	2.2 × 10^2^	±5.4	1.7 × 10^1^	±1.8	3.4 × 10^0^
V_B_4	3.3 × 10^2^	±2.0	1.7 × 10^1^	±1.7	3.4 × 10^0^
V_B_5	6.9 × 10^1^	±2.0	1.7 × 10^1^	±2.2	3.4 × 10^0^
V_B_6	6.1 × 10^2^	±3.6	1.4 × 10^1^	±0.76	2.7 × 10^0^
V_B_7	4.0 × 10^2^	±3.6	2.8 × 10^1^	±9.7	5.7 × 10^0^
V_C_1	7.6 × 10^1^	±3.0	1.9 × 10^1^	±12	3.9 × 10^0^
V_C_2	3.7 × 10^2^	±9.4	2.9 × 10^1^	±5.4	5.8 × 10^0^
V_C_3	1.3 × 10^3^	±14	1.3 × 10^1^	±1.4	2.6 × 10^0^
V_C_4	1.4 × 10^3^	±1.2	1.6 × 10^1^	±1.3	3.1 × 10^0^
V_C_5_E_	9.3 × 10^2^	±1.5	1.2 × 10^1^	±0.98	2.5 × 10^0^
V_C_5_B_	4.5 × 10^2^	±2.0	1.4 × 10^1^	±0.76	2.8 × 10^0^
V_C_6_E_	3.3 × 10^2^	±4.0	1.3 × 10^1^	±1.1	2.5 × 10^0^
V_C_6_B_	1.0 × 10^3^	±5.3	1.4 × 10^1^	±1.9	2.7 × 10^0^
PCV	1.8 × 10^2^	±1.1	1.6 × 10^1^	±1.7	3.1 × 10^0^

The length, *l*_n_, and inner diameter, *d*_n_, of each wing vein (measured via ImageJ) are listed.

**Table 2:** Surface and contact areas for *D*. *melanogaster* wing membrane cells.

Wing membrane cell	Surface area: *A*_S_ [μm^2^]	Contact area: *A*_C_ [μm^2^]
M1	1.7 × 10^4^	2.0 × 10^3^
M2	7.3 × 10^4^	4.4 × 10^3^
M3	3.2 × 10^5^	1.6 × 10^4^
M4	5.8 × 10^5^	1.3 × 10^4^
M5	5.9 × 10^5^	8.4 × 10^3^
M6_E_	5.2 × 10^5^	4.2 × 10^3^
M6_B_	2.2 × 10^5^	5.7 × 10^3^
M7	8.0 × 10^5^	6.2 × 10^3^

The surface area (*A*_S_ = 2*A*_m_, where *A*_m_ is the area of each side of a membrane) and contact area (*A*_C_ = Σ*πl*_n_*d*_n_/2) of wing membrane cells in *D*. *melanogaster* are listed. The lengths, *l*_n_, and diameters, *d*_n_, of the surrounding veins for each membrane cell are detailed in Table 1.

### Flow rate calculation

We modelled the haemolymph flow within the wing veins of the fruit fly as Poiseuille flow within a network of cylindrical pipes, which were two-dimensionally arranged. In *Anopheles gambiae*, vein diameters and flow velocities are observed to be of the order of 10^−6^ m and 10^−4^ m/s, respectively [2]. The haemolymph viscosity, *μ*, is 1.3 × 10^−3^ Pa·s, as measured in *D*.*melanogaster* at 22 °C [16]. The density is measured as 1.02 × 10^3^ kg/m^3^ at 22 °C in *Manduca sexta* [17]. Consequently, the Reynolds number for this phenomenon is of the order of 10^−5^, indicating viscous flow. For density values, we substituted those from other insect species categorised in the Holometabola superorder, to which the fruit fly belongs. The density value does not influence our calculation of the volumetric flow rate and pressure loss within the veins but was used for approximating Reynolds number of the phenomenon, as mentioned above. We considered haemolymph in the adult fruit fly as a Newtonian fluid because haemolymph in the adults of moths, classified as Holometabolan insects, is known to exhibit Newtonian behaviour [18].


$$\Delta p_{\text{n}}=\frac{128\mu l_{\text{n}}}{\pi d_{\text{n}}^{\;4}}q_{\text{n}},$$ (1)

In Poiseuille flow, pressure loss in each wing vein is defined as with the volumetric flow rate *q*_n_. We set the hydraulic resistance *R*_n_ = 128*μl*_n_/*πd*_n_^4^, which is a constant value depending on both vein geometry and the viscosity of the fluid. Therefore,

$$\Delta p_{\text{n}}=R_{\text{n}}q_{\text{n}}.$$ (2)

For each wing vein, the constant value of the hydraulic resistance is known, but the pressure loss and flow rate in this equation are unknown. We established simultaneous equations, corresponding to Ohm’s law, for all wing veins in the network as a hydraulic resistor system.

**Fig 2:**
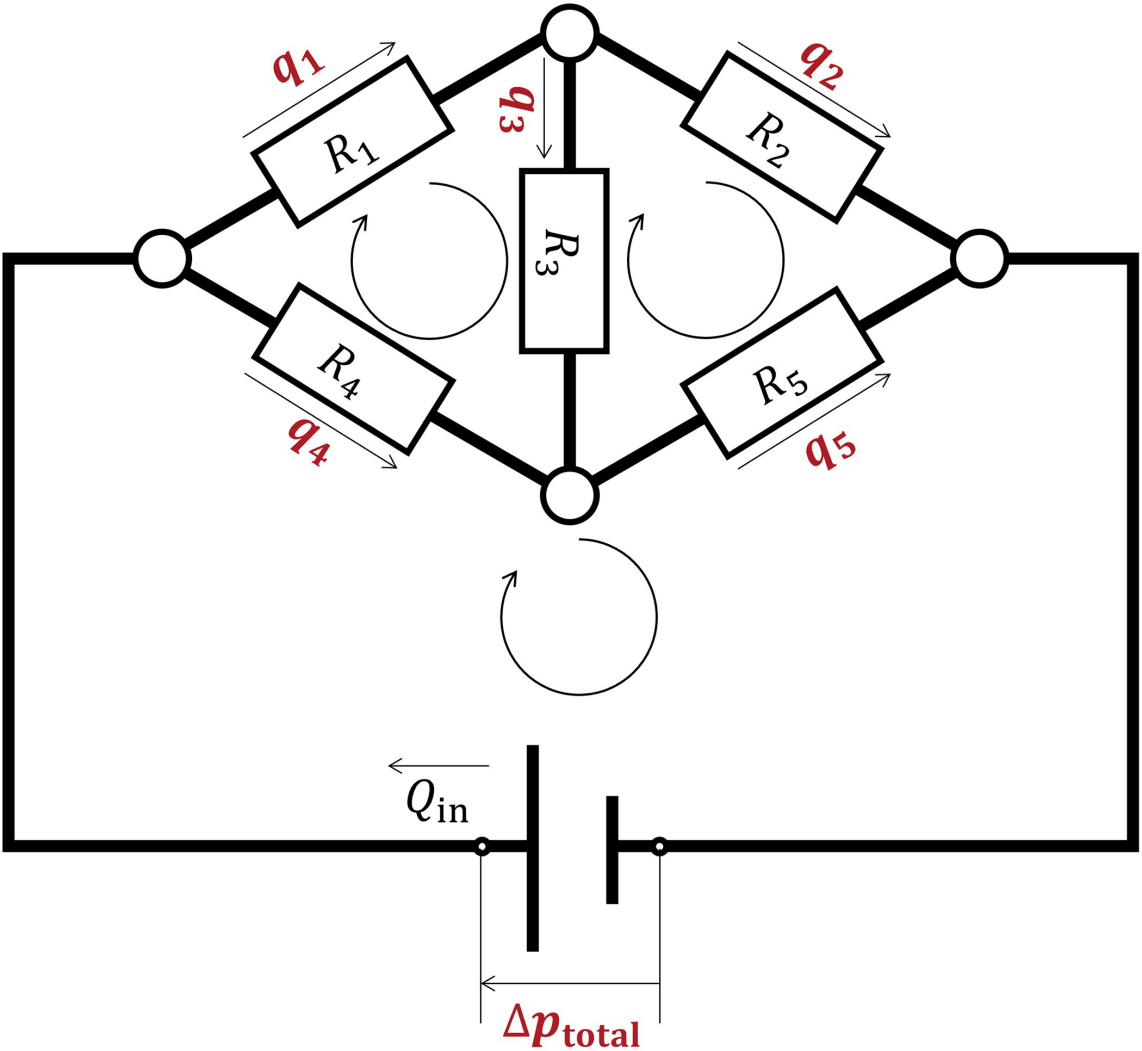
Illustration of the numerical modelling of a fluidic circuit with five resistors and three closed loops. The white circles denote nodes where fluid mass is conserved. The white rectangles denote the hydraulic resistors. The straight arrows indicate the assumed flow directions. The looped arrows denote the closed loops of the circuit whose sums of pressure losses are zero. Red physical quantities are unknown values in our calculation to calculate in the wing vein network, whereas black ones are known.

We can define a matrix by using the set of equations for the venous network based on conservation laws of mass and energy, similar to Kirchhoff’s laws of current and voltage. At any node in the network, the incoming volumetric flow rate, *q*_in_, and outgoing flow rate, *q*_out_, satisfy mass conservation: *q*_in_ = *q*_out_. Over any closed loop in the network, the sum of pressure losses in its member veins satisfies energy conservation: ΣΔ*p*_n_ = Σ*R*_n_*q*_n_ = 0. Over a closed loop with the pressure sources, the sum of pressure losses equals the source pressure, and our vein circuit model has only one source in total. Therefore, a closed loop with the pressure source in our model satisfies ΣΔ*p*_n_ = Σ*R*_n_*q*_n_ = Δ*p*_total_, where Δ*p*_total_ is the total pressure loss. For example, when we consider a hydraulic circuit with five resistors and three closed loops shown in Fig 2, the set of equations yields the following matrix form based on the conservation laws

$$\left(\begin{array}{ccc} R_{1}+R_{3}+R_{4} & -R_{3}\; & 0\\ -R_{3}\; & R_{2}+R_{5}+R_{3} & 0\\ -R_{4}\; & -R_{5}\; & -1 \end{array}\right)\cdot \left(\begin{array}{c} q_{1}\\ q_{2}\\ \Delta p_{\text{total}} \end{array}\right)=\left(\begin{array}{c} R_{4}Q_{\text{in}}\\ R_{5}Q_{\text{in}}\\ -(R_{5}+R_{4})Q_{\text{in}} \end{array}\right),$$ (3)

where *Q*_in_ is the inflow rate. This matrix has *Ax* = *y* notation, where matrix *A* contains the known values of hydraulic resistance of the veins and 0 or −1 denote the inclusion of unknown Δ*p*_total_ in each loop, vector *x* contains the unknown values of volumetric flow rates and Δ*p*_total_, and *y* contains known pressure loss expressed by known volumetric inflow rate and hydraulic resistance. The number of equations should correspond the number of unknown variables to express the matrix. The interdependent variables are combined (e.g., *q*_3_ = *q*_1_ − *q*_2_) to minimise the number of equations. We established the matrix equations for wing vein network model of the fruit fly following the same concept, with known *R*_n_ and *Q*_in_ and unknown *q*_n_ and Δ*p*_total_. We solved the linear matrices in MATLAB (MathWorks, Natick, MA, USA) with double precision. This solution has been commonly used in the field of microfluidic networks [19, 20] including microvascular network systems [21].

We confirmed that our numerical method could derive flow distributions and pressure losses in simple hydraulic circuit models, as they satisfy both conservation laws of fluid mass and energy. The following flow parameters were consistent, being independent from the absolute value of inflow rate: the individual flow rates normalised by the inflow rate and relative changes in overall pressure loss in the network due to vein removal or rearrangement. We tested another solving method used in pipe network analyses, the Hardy–Cross method [22, 23], and confirmed that our solution of flow rates and pressure losses contain less than 0.7% of differences, which originated from dependence on solution convergence in the Hardy–Cross method.

We fixed the inflow rate and outflow rate, *Q*_out_, of the venous network of the forewing of *D*. *melanogaster* at the boundary; *Q*_in_ = *Q*_out_ = 5.2 × 10^2^ μm^3^/s. This value from was estimated from a previous report on the flow velocity and diameter at the forewing inlet of another Dipteran species, *A*. *gambiae* [2]. We set the entrance and exit of haemolymph flow at the anterior and posterior wing base as illustrated in Fig 1 and assumed that haemolymph fills all the wing veins of fruit flies, based on the previous reports on other Dipteran species [1, 2]. We obtained the *R*_n_ values of respective veins from the fluid characteristics and the measured vein geometries. We ignored minor losses excluding frictional loss because it is approximately 10^5^ times larger than others, referring to the present Reynolds number [24]. The calculated values of hydraulic resistance and pressure loss in the veins may be in the range of 0.57–1.9 times of the true values owing to the limit of spatial resolution of image analysis.

### Network modelling for shifting connections of the PCV

We used the vein network model with the measured geometry, as explained above, to demonstrate the effects of the PCV’s presence by virtually comparing haemodynamics in the presence and absence of this vein. In contrast, to investigate the positional effect of the PCV’s connections, simplifying the vein network model based on the measured vein geometry was necessary.

**Fig 3:**
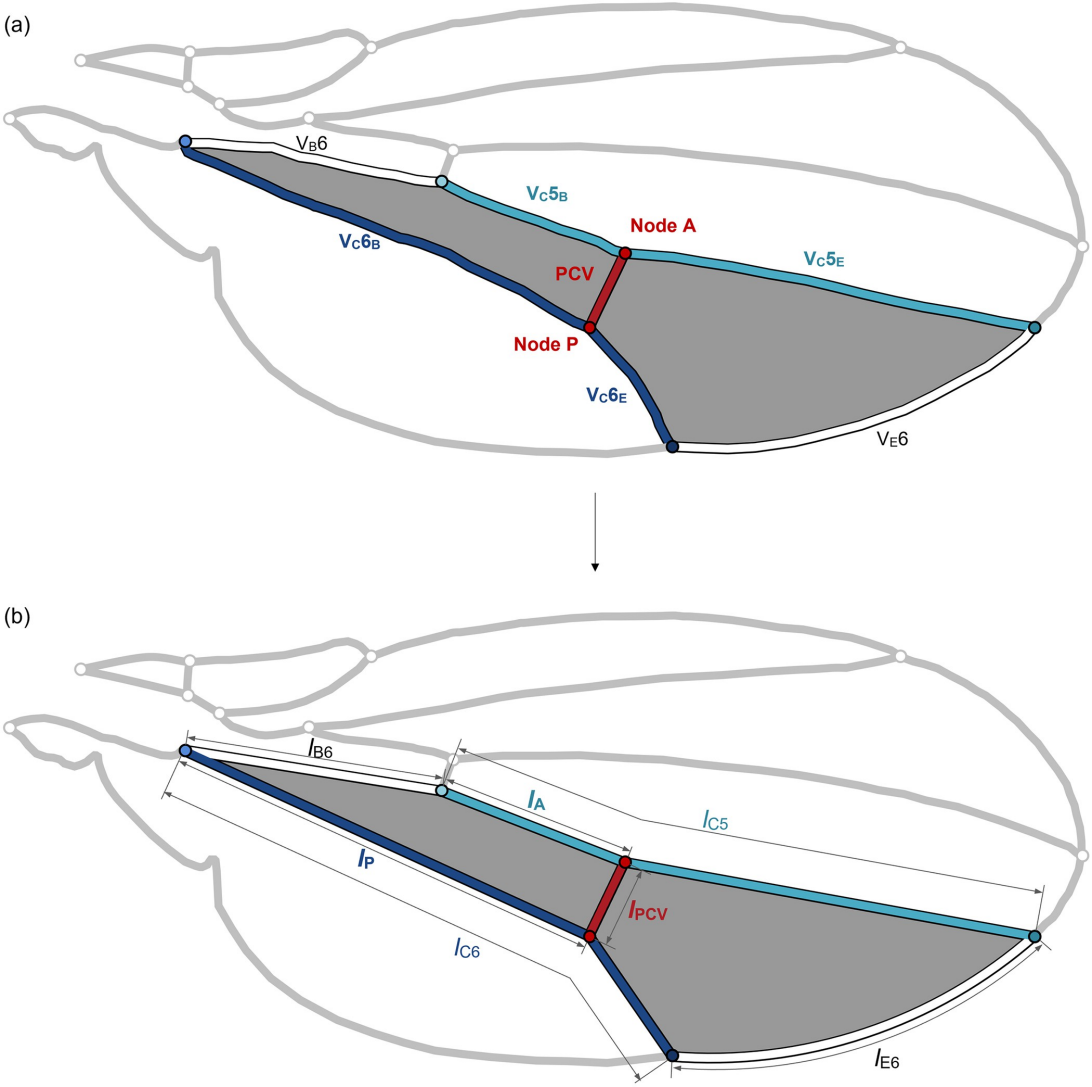
Schematic modelling of the wing membrane cells, M6, divided by the posterior cross vein (PCV). (a) Actual membrane cells. (b) Schematic model of the membrane cells. The membrane cell containing the PCV is surrounded by the edge vein, V_E_6; base vein, V_B_6; and anterior and posterior connecting veins, V_C_5 and V_C_6, respectively. The base vein and segments of connecting veins are represented by straight-line segments connecting their ends. The red circles labelled “Node A” and “Node P” indicate the anterior and posterior connections of the PCV, respectively, separating these connecting veins. The lengths of their base-side segments, V_C_5_B_ and V_C_6_B_, are respectively designated as the variables *l*_A_ and *l*_P_.

We ascertained the effect of its position within a wing membrane cell, where this vein is located, on the ratio of the areas by virtually changing the position of the PCV connections. The anterior and posterior connections of this vein respectively divide the connecting veins in the middle, V_C_5 and V_C_6, referred to as Node A and Node P as shown in Fig 3a. We virtually shifted these connections along these veins. Corresponding to the positions of the connections, we derived the lengths of the veins surrounding the membrane cells separated by the PCV and the areas of these membrane cells. These geometric parameters were then employed to calculate the values of *A*_S_ /*A*_C_ for the membrane cells during the shifting of connections.

The simplification of the geometry of membrane cells separated by the PCV was necessary to avoid an excessive degree of complexity for the aforementioned analysis. The vein segments surrounding the membrane cells were substituted by a combination of straight-line segments and an arc, as illustrated in Fig 3b. The ends of each vein segment were connected by a straight-line segment, except for the edge vein, which was fitted with an arc intersecting both ends of the actual edge vein. This arc was implemented to minimise modelling errors in the vein length and the area of the membrane cell. The PCV was defined as the straight line linking the shifted connections. Certain combinations of connection points could not define the PCV because the straight line between them intersected other veins. These combinations were excluded from the present analyses. The coordinates of the vein ends were measured using ImageJ software, and the membrane area and vein lengths related to the simplified membrane cells were calculated based on the measured coordinates. The simplified model contains errors in the lengths of vein segments and the areas of membrane cells, but these errors did not exceed 5%. The calculated flow rates include errors less than 3% owing to the errors in the vein lengths after model simplification compared with those in the non-simplified model. The overall pressure loss in the simplified model was calculated 1.8% smaller than the non-simplified model.

We defined the lengths of the base-side segments of these connecting veins, *l*_A_ and *l*_P_, as variables indicating the positions of Nodes A and P, as shown in Fig 3b. We varied both *l*_A_ / *l*_C5_ and *l*_P_ / *l*_C6_, ranging from 0.001 to 0.999 in increments of 0.001, where *l*_C5_ and *l*_C6_ represent the lengths of the anterior and posterior connecting veins as shown in Fig 3b. In this simplified model, the actual PCV position corresponded to *l*_A_ / *l*_C5_ = 0.322 and *l*_P_ / *l*_C6_ = 0.754, with respective errors of 1.1% and 0.10% due to the simplification.

## Results and discussion

### Division of a wing membrane cell by the PCV results in maximum *A*_S_ /*A*_C_

**Fig 4:**
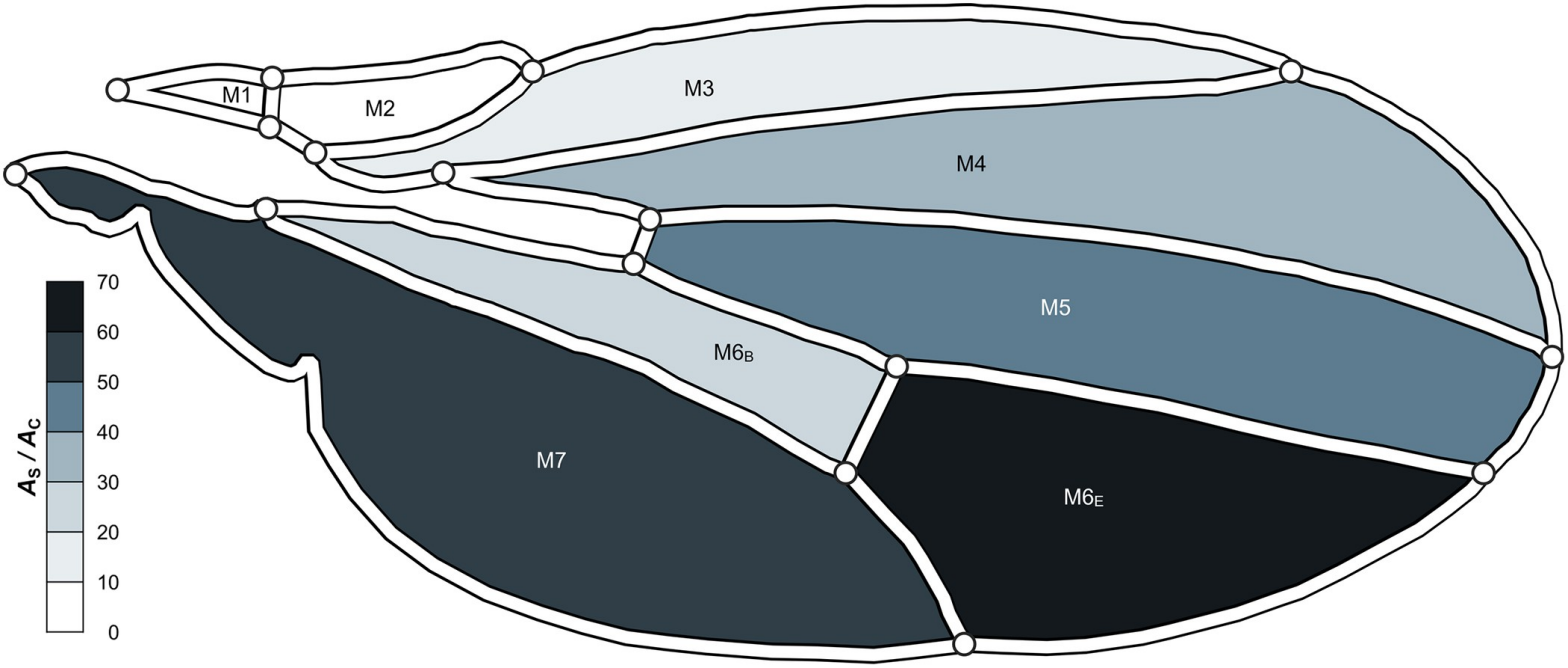
Distribution of *A*_S_ / *A*_C_ values of the wing membrane cells. The membrane cells are coloured based on the values of *A*_S_ / *A*_C_. High values are represented by dark colours, and low values by light colours.

We characterised the geometry of wing membrane cells by determining the ratios of their surface area to the contact area with their surrounding veins, denoted as *A*_S_ /*A*_C_. These ratios were relatively low in the anterior region and high in the posterior region, as shown in Fig 4. In the anterior cells, M1–M4, the surface area exceeded the contact area by an average factor of 16. By contrast, the remaining membrane cells exhibited a surface area 46 times larger than the contact area on average. The PCV divided the sixth membrane cell, M6, into two segments with individual *A*_S_ /*A*_C_ values, as depicted in Fig 4. Notably, the separated cell on the base side obtained 50% of the ratio of the unseparated sixth cell. Conversely, the other divided cell exhibited a 38% increase in the ratio, with its surface area being 64 times larger than its contact area. This ratio of the areas was the largest among all membrane cells.

**Fig 5:**
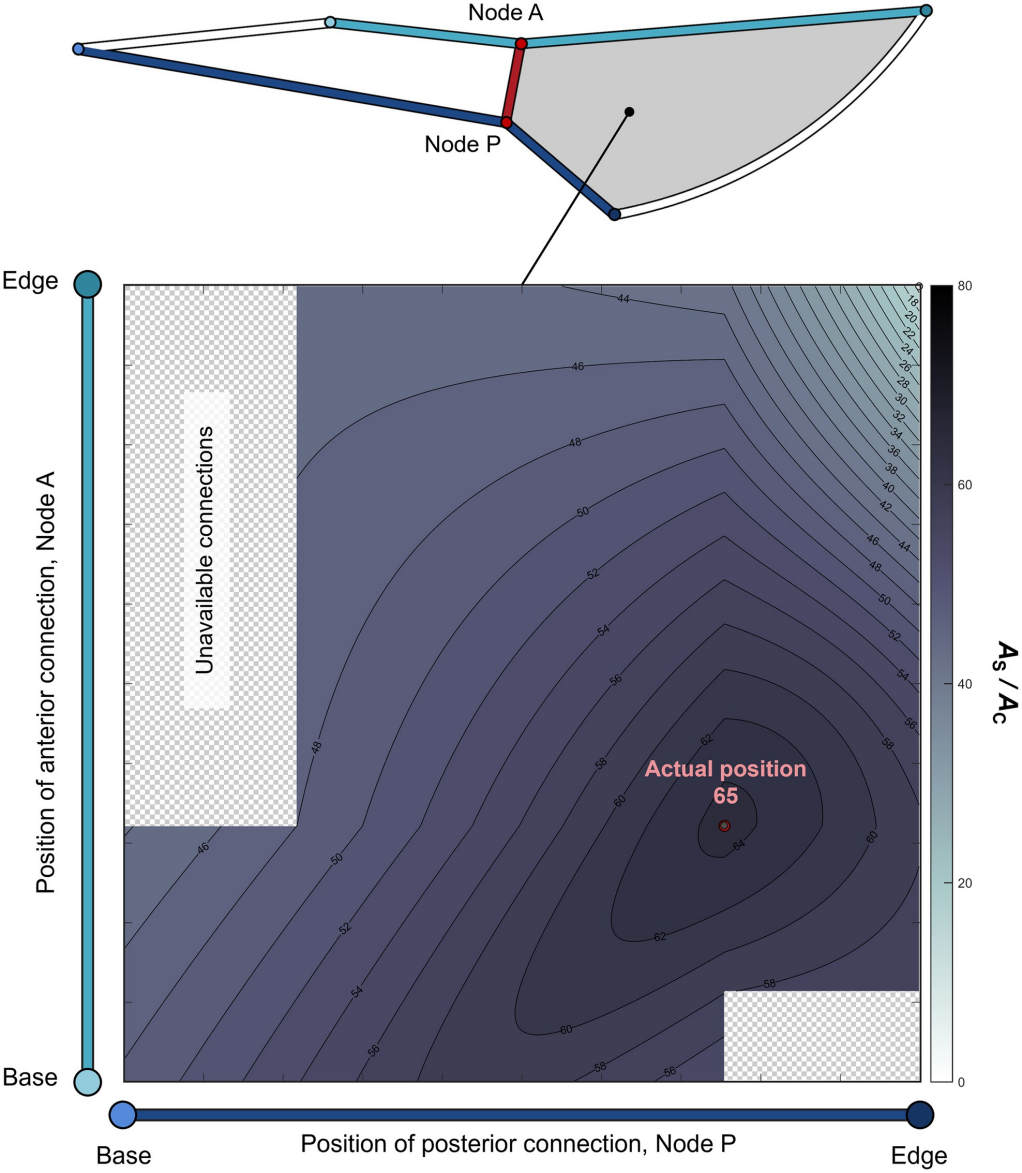
Influence of the posterior cross vein (PCV) position on *A*_S_ / *A*_C_. Variation in *A*_S_ / *A*_C_ values of the separated membrane cells on the edge side. The light blue vertical and dark blue horizontal axes indicate the positions of the PCV connections on the anterior and posterior connecting veins, Nodes A and P, respectively. High values are represented by dark colours, and low values by light colours. The red circle captioned “Actual position” shows the actual positions of the connections in the simplified model and the number below is the *A*_S_ / *A*_C_ value at the actual positions. The maximum is obtained at the position of the black dot overlying the red circle. The minimum value is obtained at the position of the white dot in the upper-right corner of the colourmap.

This area ratio in the edge-side cell was maximised by the connecting positions of the PCV within the sixth membrane cell. We examined *A*_S_ /*A*_C_ in the edge-side cell with the rearranged PCV connections and present the relationship between this ratio and the connecting positions as a colormap in Fig 5. The red and black dots coincide at the same point, which respectively indicate the actual positions and the positions with the maximum *A*_S_ /*A*_C_. This shows that this edge-side membrane cell has a geometric characteristic wherein the surface area relative to the contact area is maximised owing to the location of division by the PCV.

We considered that the ratio of the areas influences the balance between seepage flow into the wing membrane and evaporative flow from the membrane. Water in the haemolymph within these veins is generally considered to seep into the wing cuticle [25] before subsequently evaporating [7, 25]. To satisfy mass conservation of water between the incoming seepage flow and the outgoing evaporation flow in the membrane, *v*_in_ *A*_in_ = *v*_out_ *A*_out_, where *v*_in_ and *v*_out_ respectively represent the velocities of the seeping inflow and evaporative outflow, and *A*_in_ and *A*_out_ respectively represent the cross-sectional areas of these flows. Thus, the ratio of these areas determines the required ratio of flow velocities: *v*_in_ */ v*_out_ = *A*_out_ */ A*_in_. The areas of evaporation and seepage can be respectively substituted by the surface and contact areas with the veins; *A*_S_ /*A*_C_ *= A*_out_ */ A*_in_ = *v*_in_ */ v*_out_. Therefore, the ratio of areas, *A*_S_ /*A*_C_, indicates how much faster the seepage flow velocity should be than the evaporation flow velocity to conserve the mass of water in the membrane cell.

When the evaporative velocity *v*_out_ is shared among all membrane cells, the ratio of areas indicates the seepage velocity *v*_in_ required to avoid water accumulation or desiccation of the membrane cell, based on this seepage-evaporation model. The two most posterior wing membrane cells, M6_E_ and M7 in Fig 4, had area ratios 2.6 times larger than the average of the remaining. This implies that these membrane cells require seepage velocities that are 2.6 times higher than those of other cells to avoid drying out. This distribution of values of *A*_S_ /*A*_C_ may cause varying mechanical properties across the wing. Water content in the wing is feasibly related to the mass of the cuticle and the moment of inertia to locomote the wing. It is also known to influence other mechanical properties, according to reports where desiccation of the insect wing increases wing stiffness [7] and decreases damping ratio [8].

### Influence of the PCV on internal haemolymph flow in the wing vein network

**Fig 6:**
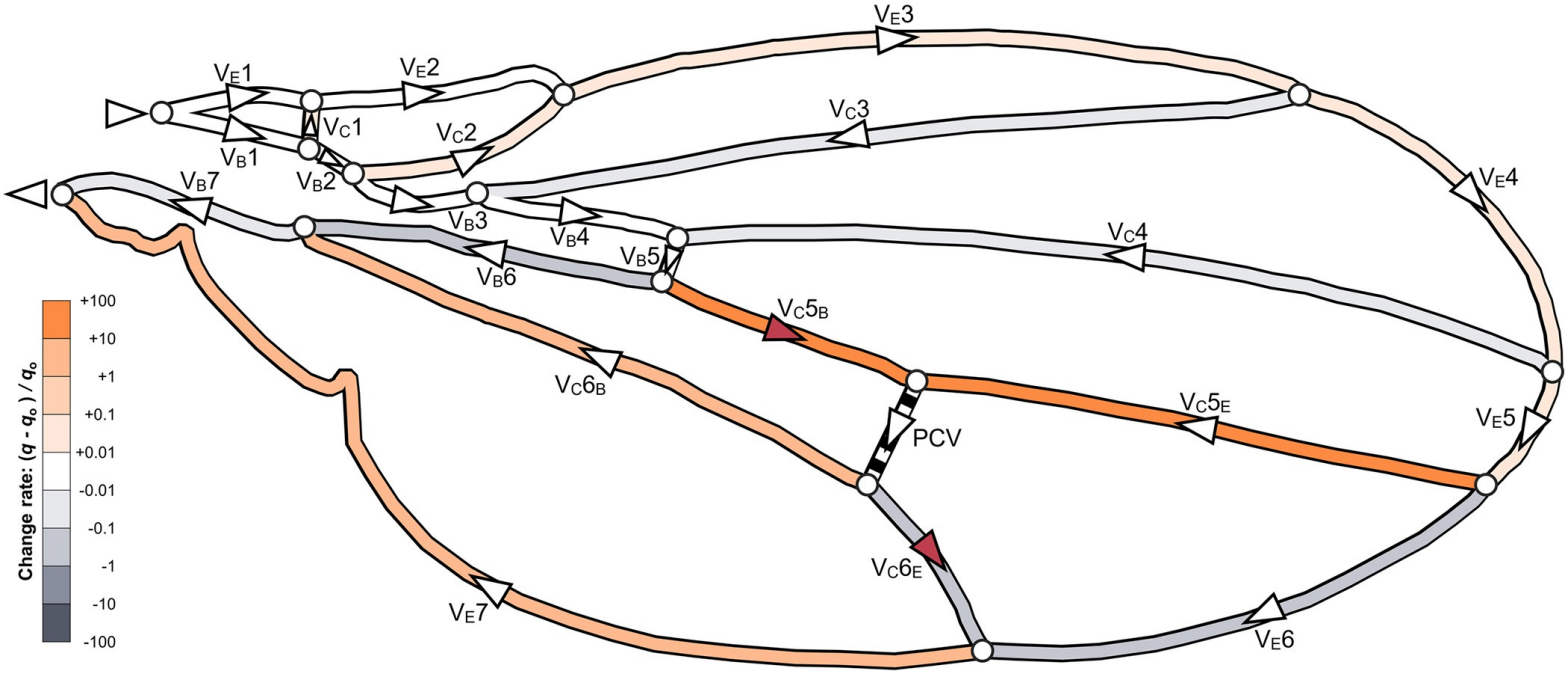
Changes in the flow rate and flow directions owing to the presence of the PCV. The colours of the wing veins are based on their values of Δ*q* /*q*_o_, where Δ*q* = *q* − *q*_o_, and *q* and *q*_o_ are the flow rates in the presence and absence of the PCV, respectively. Orange indicates an increase, and grey a decrease in the values from those in the absence of the PCV. The white triangles on the veins denote the directions of the flow, which are analytically obtained, and the red triangles denote the changed flow direction owing to the PCV’s presence. The colour bar is logarithmic.

We examined how the presence of the PCV influenced the internal haemolymph flow under steady-state conditions with a constant inflow rate of *Q*_in_ = 5.2×10^2^ μm^3^/s by comparing haemolymph flow in its presence to that in its absence. In the connecting vein that is anteriorly adjacent to this vein, V_C_5, haemolymph flow rates were 16 times and 54 times more in its edge-side and base-side segments, respectively, compared with those in the absence of this vein, as indicated in strong orange in Fig 6. The flow direction is towards the edge from the base in its base-side segment, which opposes that in the absence of this vein. Both segments of this anterior vein supplied a total of 0.32*Q*_in_ of haemolymph flow rate to the PCV, which was drawn from the edge and base veins. The flow direction also changed in the edge-side segment of the posteriorly adjacent connecting vein, V_C_6, as shown in Fig 6. Haemolymph flow in the PCV was distributed back to the edge and base through both segments of this posterior vein. Consequently, some of haemolymph bypassed the sixth pair of the edge and base veins, V_E_6 and V_B_6, causing respective decreases of 69% and 28% in flow rate in these veins, as shown in Fig 6. The total decreased flow rate was the same as the flow rate in the PCV parallel to this vein pair in our topological model in Fig 1b. A 20% decrease was observed in the overall pressure drop between the entrance and exit of the network in the presence of this vein, whereas this pressure drop was 4.0×10^2^ Pa in its absence.

Our calculated flow rate distribution in the wing vein network of the fruit fly was conceivable based on the resultant flow paths observed in the actual insect wings. Our computational flow path in the vein network represents the characteristics of the experimentally reported ones in insects in Diptera order or some other insect species, such as Lepidoptera [1, 2]; haemolymph flows in the vein series along the wing edge and base from the anterior entrance towards the posterior exit, and most flow directions in the connecting veins are from the wing edge to the base [1, 2]. The calculated value of the pressure loss for the flow rate distribution is feasible based on the size comparison of the accessory pulsatile organs with a blood-sucking pump in mosquito belonging to the Diptera order as well as the fruit fly. This suction pump was simulated to generate a time-averaged pressure of 0.38 kPa approximately to overcome pressure loss in its proboscis during continuous sucking and has 232 μm of length and 0.18 nL of the rest volume [26]. Our estimation of the pressure loss in the wing veins of the fruit fly was 16% smaller than the pressure output of the mosquito pump, and its pulsatile organ has 35% smaller length and 64% smaller volume approximated from the cross-sectional images of the organ [27, 28]; they are on the same order of magnitude. Therefore, our calculated results indicate agreement with the previous reports on physiology in insects from comparative viewpoint.

**Fig 7:**
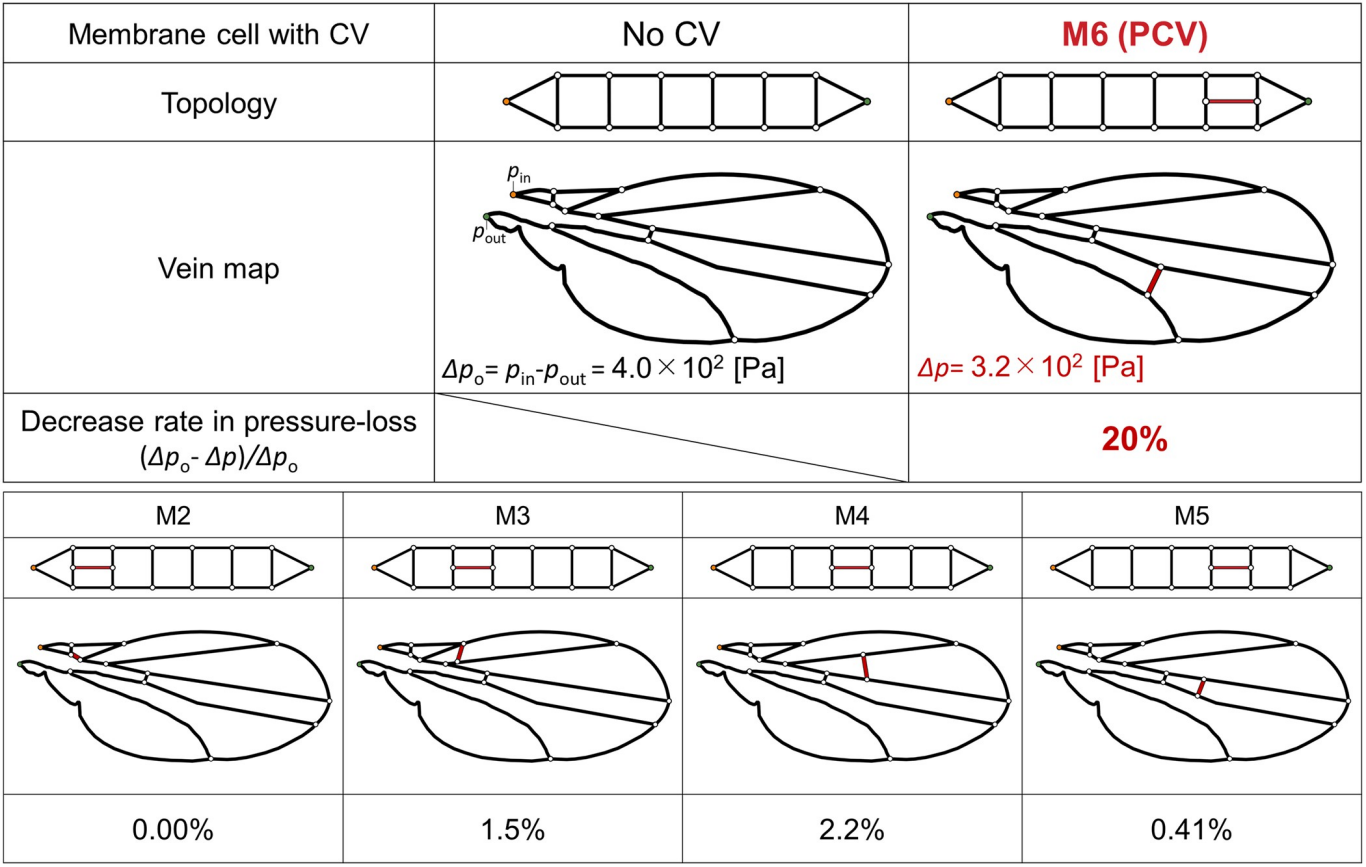
Overall pressure loss in the wing with cross-veins. “Membrane cell with CV” indicates the membrane cell where the cross vein is located. “Topology” and “Vein map” show the locations of the corresponding cross veins in the topological venation model and the actual venation as the red veins. “Decrease rate in pressure-loss” represents the decrease in the overall pressure loss due to the presence of each cross vein. “CV” in this figure denotes “cross vein”.

The local pressure drop within a vein is proportional to its flow rate according to Eq (2), and the total pressure loss is equal to the sum of the local pressure drops within the edge or base vein series. We considered the decrease in the total pressure loss to be a result of the decreased flow rate in the sixth vein pair via bypassing flow through the PCV. However, if a decrease in total pressure loss was caused by a haemolymph flow bypassing the edge and base veins, a cross-vein topologically parallel to them would have the same effect. We were interested in the pressure-loss reducing effect of other cross-veins that substitute for the PCV by linking two other connecting veins and being parallel to the edge-base vein pairs, as shown in Fig 7. We virtually arranged the cross-veins within their corresponding wing membrane cell to satisfy the requirement of maximising *A*_S_ /*A*_C_ on the edge-side divided cell, as same as the PCV. We determined the position of each virtual vein through the shifting of its connections after a geometric simplification of each membrane cell, and its length was determined by the connecting positions. Its inner diameter was adjusted to equalise the value of *l*_n_/*d*_n_^4^ to that of the PCV which is proportional to the local pressure loss within the vein according to Eq (1). However, these alternative veins did not reduce the pressure loss by more than 3%, as shown in Fig 7; the actual vein network with the PCV obtained the largest decrease in total pressure loss.

**Fig 8:**
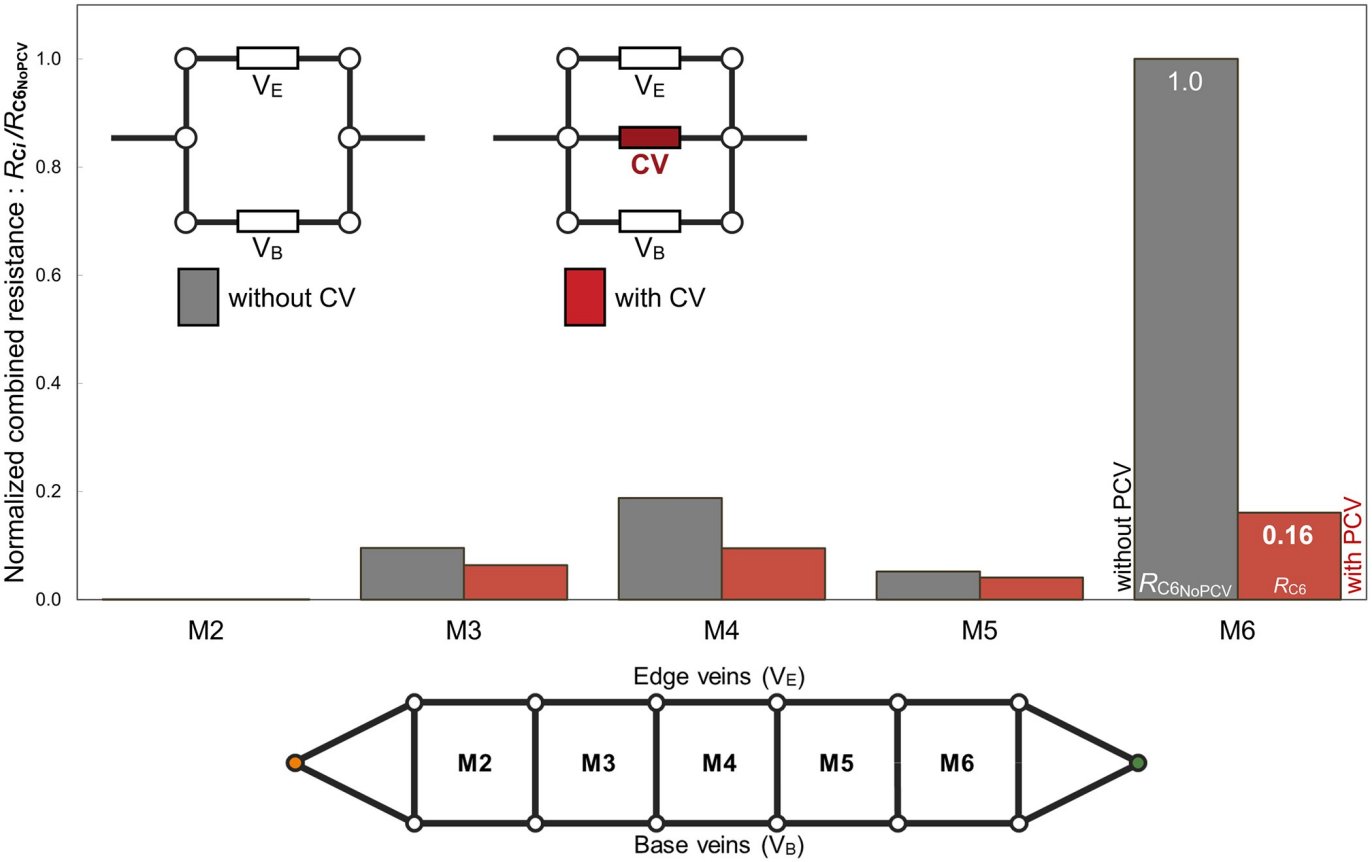
Normalised combined resistance of a pair of edge and base veins with or without a cross vein in each membrane cell. The vertical axis shows the combined resistance of a group of veins that is normalised by that of the sixth pair of edge and base veins without the PCV. Grey and red bars respectively denote normalised combined resistances of vein pairs without and with the alternative cross veins in corresponding membrane cells. “CV” in this figure denotes “cross vein”. For the sixth membrane cell, M6, “CV” corresponds to the PCV.

We treated paired edge and base veins as paralleled hydraulic resistors and calculated their combined hydraulic resistance. The sixth edge-base vein pair exhibited the highest combined resistance among all pairs, being over 5 times larger than the second largest one, as indicated by the grey bar on M6 in Fig 8. However, the combined resistance of the sixth vein pair and the PCV equalled 16%, as shown by the red bar on M6 in Fig 8. We concluded that this vein functions as a hydraulic resistor parallel to the vein pair with the highest resistance and causes the largest decrease in overall pressure loss to generate haemolymph flow.

The pressure loss within the vein network determines the power consumption via haemolymph transport, impacting the overall energy budget of the insect. It decreases by 20%, down to 1.6×10^−13^ W in the fruit fly, with the overall pressure loss improved owing to the PCV presence, evaluated from the product of the overall pressure loss and the flow rate at the entrance. The PCV can be one of results from evolutionary adjustment of the venous network to improve the power consumption owing to haemolymph transport. It may explain the reason for the common presence and location of this cross-vein in the wings of all species in Drosophila genus.

Our results provide insight into the modification of microfluidic networks. The minimisation of the power consumption owing to frictional loss improves energy efficiency of the fluid transport system. Some microfluidic network design can employ the wing vein network structure, such as fluidic radiators, in micro-electric devices and micro-channels of lab-on-chip devices [19]. From a cardiovascular engineering perspective, the venous network can inspire bypassing surgery technique for microvascular networks to reduce their internal frictional stress.

## Conclusion

Our results revealed the fluid mechanical effects of the PCV on haemolymph circulation network of the wing veins. A membrane cell is divided by the PCV, and its edge-side divided portion obtains the maximum ratio of surface area and contact area with the surrounding veins and the actual connecting positions of this vein. The setting of this vein in the wing vein network is topologically parallel to a pair of veins aligned on the edge and base have the highest hydraulic resistance. This results in changes in the haemolymph flow pattern and a decrease in the pressure loss within the network from the entrance to the exit by 20%, although other cross veins substituting for the PCV exhibit smaller decreases in pressure loss. We uncovered the effect of this vein on the haemolymphatic dynamics via numerical analyses of the wing vein network based on its analogy with a hydraulic circuit.
